# Adolescent Use and Perceptions of JUUL and Other Pod-Style e-Cigarettes: A Qualitative Study to Inform Prevention

**DOI:** 10.3390/ijerph18094843

**Published:** 2021-05-01

**Authors:** Kimberly G. Wagoner, Jessica L. King, Amir Alexander, Hollie L. Tripp, Erin L. Sutfin

**Affiliations:** 1 Department of Social Sciences and Health Policy, Wake Forest School of Medicine, Medical Center Boulevard, Winston-Salem, NC 27157, USA; esutfin@wakehealth.edu; 2 College of Health, University of Utah, Salt Lake City, UT 84112, USA; jess.king@utah.edu; 3 Department of Biostatistics and Data Science, Wake Forest School of Medicine, Medical Center Boulevard, Winston-Salem, NC 27157, USA; aaalexan@wakehealth.edu; 4 Department of Psychiatry, University of Pennsylvania Perelman School of Medicine, Philadelphia, PA 19104, USA; Hollie.Tripp@Pennmedicine.upenn.edu

**Keywords:** e-cigarettes, vaping, social influences, environmental influences, adolescent, qualitative

## Abstract

JUUL, a discrete pod-style e-cigarette, popular among adolescents, delivers high levels of nicotine. Limited research has assessed social and environmental influences that contribute to use of JUUL and other pod-style devices. We examined how these factors, as well as individual characteristics, shape adolescent use. Twenty-nine middle and high school students participated in six focus groups in June 2019 (58.6% female, 65.5% White, 27.6% Hispanic). Groups were stratified by e-cigarette use status and grade to understand perceptions and experiences among groups. Transcripts were coded using thematic analysis for individual, social, and environmental factors contributing to use. Users (*n* = 13) described their first experience with JUUL as mostly negative, mentioning reactions such as burning in the throat, coughing, wheezing, and headaches. Despite a negative first experience, stress relief and addiction were mentioned as reasons for continued use. Users and non-users identified vaping as a source of disruption to their daily life. Social factors included peer and parental influences, lack of support for quitting, and accessibility. Environmental factors included contrasting messages about long- and short-term health effects of e-cigarettes, as well as a lack of school vaping policy enforcement, health education, medical screenings, and cessation resources. Findings highlight the complex social system that influences adolescent e-cigarette use and have important implications for school and community responses. Strategies to prevent or reduce use may include reviewing existing school tobacco policies, providing counseling and cessation resources, training staff, and increasing knowledge through public education campaigns.

## 1. Introduction

E-cigarettes are the most common form of tobacco used among middle and high school students in the United States [1,2]. In 2019, 27.5% of high school students and 10.5% of middle school students, representing over 5 million adolescents, reported current e-cigarette use [1,3]. Contributing to this increase is the latest generation of pod-style e-cigarettes. One of the most popular brands, JUUL, resembles a computer flash drive and contains e-liquid made of nicotine salts and benzoic acid, allowing nicotine to be delivered at high concentrations without harshness and creating a pathway for nicotine addiction [4,5].

Although JUUL claimed it was designed for adult smokers as a “satisfying alternative to smoking cigarettes” and did not target those underage [6], JUUL’s initial marketing focused on social media where it effectively permeated social networks on Instagram, Twitter and YouTube [7,8,9]. These efforts successfully reached adolescents, as studies showed those ages 15 to 17 were more likely to use JUUL than adults [10,11,12]. Contributing to JUUL’s popularity are youth-appealing flavors [13,14], high nicotine content [15], and discreet design, making it easy to conceal from parents and teachers [16,17,18].

Few studies have assessed the broader contexts that contribute to why, how, and under what conditions JUUL is used by adolescents. Social ecological approaches recognize that individuals partake in substance use because of a complex social system that includes societal level influence such as broad population-level trends and public policy, influencers closer to the adolescent such as families and peers, as well as individual perceptions and personal characteristics [19]. Therefore, understanding how factors at varying levels influence use is needed to inform prevention efforts and intervention development. The few studies that have assessed the broader contexts of adolescent e-cigarette use have shown that a more pro e-cigarette environment is associated with adolescent e-cigarette use. For example, density of e-cigarette retailers around schools is associated with ever and past month student use [20] and marketing targeting adolescents has contributed to use among this population [5,9]. Additionally, the school environment can influence student e-cigarette use. For example, students attending schools with moderate to high lifetime e-cigarette use among its student body had two to four times higher odds of current e-cigarette use compared to students attending schools with a low prevalence [21]. 

To gain a better understanding of how these contexts influence use, we conducted focus groups with middle and high school students. Focus groups allowed for open-ended questions to explore attitudes and perceptions, while also using probes to gain more detail about contexts associated with use.

## 2. Materials and Methods

### 2.1. Participants

#### School and Student Recruitment 

We partnered with a North Carolina school district to recruit schools. Upon consultation with district administrators, two high schools and one middle school representing diverse geographical and socioeconomic areas were invited to participate. 

Letters were sent to school administrators to gauge interest in participation. The study team met with school administrators to further discuss the study and identify best methods to inform parents and recruit students. Recruitment included flyers posted at school and announcements made through school communication channels. Parents were informed via physical and digital flyers, which included brief information about the study, as well as how to opt their child out of the study. Flyers directed interested parents and students to a website containing study information, frequently asked questions and answers, and a link to an eligibility screening survey. Interested students completed the screener, which assessed awareness of JUUL and other e-cigarettes, and provided information such as, age, grade, and contact information. 

Adolescents who completed the screener and were not opted out by a parent were contacted by the study team regarding eligibility and next steps for the focus groups. An informed consent form was emailed to parents of eligible students; parents signed the consent form using DocuSign. Once obtained, the student was notified of the date, time, and location of their assigned focus group. 

Eligible students who self-identified as having used an e-cigarette were assigned to a userfocus group; those aware of e-cigarettes, but reported never use, were assigned to a non-user group. Groups were separated by user status to minimize non-users’ exposure to use-related discussions. High school focus groups were divided into groups with underclassmen (freshmen/sophomores) or upperclassmen (juniors/seniors) due to potential differences in life experience and substance use exposure. 

### 2.2. Procedures

Six focus groups were conducted in June 2019. We collaborated with the Wake Forest Qualitative and Patient-Reported Shared Resource for data collection and analysis. Each group was facilitated by a trained team of three: a moderator, co-moderator, and note taker. Focus groups were held after school in private classrooms at students’ respective schools. Groups consisted of two to 10 participants. 

Prior to starting the group, the study team confirmed a parental signed consent form was on-file. Participants under 18 provided written assent of affirmative agreement to participate; those 18 and older provided written consent to participate. After assent or consent was obtained, participants completed a brief survey to obtain demographic information and tobacco use history. Upon survey completion, moderators used semi-structured interview guides to facilitate the focus groups. Focus groups were audio-taped and lasted 60–90 minutes. At the conclusion, participants received a $40 gift card, were debriefed about harms of use, and were provided cessation resources. The study protocol was approved by the Wake Forest School of Medicine Institutional Review Board.

#### Moderator Guides

Two moderator guides were developed and began with discussion of perceptions of JUUL and other e-cigarettes. Questions in the user guide focused on revealing contextual data that described why, how, and under what conditions e-cigarettes are used. The non-user guide focused on understanding resistance to use and non-users’ experiences with users and vaping.

### 2.3. Data Analysis

Survey data were entered into Excel and frequencies were calculated. Focus group recordings were professionally transcribed and reviewed for accuracy. Atlas.ti was used to store and manage the data [22]. A codebook identifying the relevant categories was created. Although the categories were pre-determined, themes were allowed to emerge, based on participant responses. Each transcript was independently coded by two trained team members [23]. Coders met periodically to resolve discrepancies. After coding, data were synthesized and results were compared by school level and user status [22].

## 3. Results

### 3.1. Sample Characteristics

Three user and three non-user focus groups were conducted. Of the six groups, one was conducted with middle school students, three with freshmen/sophomores, and two with juniors/seniors. Twenty-nine students participated, including 16 non-users and 13 ever users. Most participants were female (58.6%); 65.5% white, and 27.6% Hispanic (Table 1). Despite efforts to have separate groups for users and non-users, crossover occurred. Three students who reported no use on the screener, identified as a user during their focus group with non-users. Among users, 37.5% reported use in the past week, 25% reported use in the past month and 31.3% reported use in the past 6 months. JUUL was the preferred brand for most users (77%), while mint was the preferred flavor for over half the respondents (57.1%) followed by mango (14.3%) and crème (14.3%). Major themes that emerged from the focus group discussions are summarized in Table 2, noting similarities and differences by user groups.

### 3.2. General Perceptions

All participants reported familiarity with JUUL and e-cigarettes including Vuse Alto, Suorin, and blu. Although “JUUL” and “JUULing” were the most reported terms used to describe use, participants used other terms interchangeably, including “vaping”, “hitting” and “ripping the JUUL”. Participants expressed uncertainty when asked if JUUL is a tobacco product; however, across all groups, participants were confident that JUUL contains nicotine. Users and non-users reported that JUUL is appealing due to its novelty, discreteness and youth-friendly flavors.

### 3.3. Individual Factors 

Themes in this category included flavor use, continued use after negative first experience, perceived addiction, risk perceptions, disruption to daily life, and use as a coping strategy.

#### 3.3.1. Flavors and Impact on Use

Most participants reported that flavors are a positive attribute of JUUL. Users and non-users thought flavors contributed to students trying JUUL. One upperclassman explained, “The flavors are the thing that attracts children and teens. Adults want something that’s a little more neutral and normal, just for them”. Another user said, “…the flavors (are) more attractive. There’s fruity flavors…it attracts high schoolers because the disgusting cigarette stereotype is away, and it’s like, ‘oh, this isn’t as bad because it’s fruity,’ so it attracts kids”.

Users also reported previous use of flavors that were removed from retailers such as mango and cucumber. They reported switching to mint-flavored pods, JUUL-compatible “knock-offs”, or refilled the pods with other e-liquids. 

#### 3.3.2. Continued Use, Even after a Negative First Use Experience

Users were asked to describe the first time they used JUUL. All participants reported that their first use was by direct invitation from a peer user. The first experience with JUUL was described as mostly negative, including coughing, wheezing, and headaches. One participant spoke about their throat burning and coughing so much “I thought I was dying”. A participant in the Junior/Senior group shared a similar experience, “I was with my friend. I took a hit. As soon as it hit my throat, it was like nothing else. I was choking, I couldn’t breathe, and I was coughing so hard. But then I got used to it and now I’m a full-time addict”. 

A freshmen/sophomore user explained the first experience, “We were at a chorus concert. They gave it to me. I inhaled… I thought it was just gonna stop for me. It started burning really, really bad, and then you just start coughing”.

#### 3.3.3. Perceived Addiction

Most participants across groups reported that JUUL is addictive and they know peers addicted to it. Some users described characteristics of addiction that they had experienced including using JUUL “immediately” or “right away” after waking up. One user explained, “It’s like, before I even open my eyes, I’m reaching out to my bedside table to grab my JUUL”. Another user said she had “to use it every second of your life to feel normal”.

Some users recognized that JUUL has a higher nicotine content than other e-cigarettes and reported seeking out pods with 5% nicotine. One Junior/Senior user explained, “The Vuse only has like 4% nicotine, I think, and the JUUL has five, so that just makes it better automatically”.

#### 3.3.4. Risk Perceptions 

Participants spoke about JUUL’s potential harms relative to combustible cigarettes, perceiving JUUL as less harmful. Users and non-users discussed similar potential health effects of use including cancer and problems with their teeth and lungs. Perceived severity of health effects did not differ between user groups. Users discussed more specific acute effects than non-users including wheezing, headache, eye irritation, and nausea. Although users and non-users said the long-term health effects of use are “kind of a mystery,” perception of harm was low. One user explained, “...everyone obviously knows it’s not good for you, but there’s no REAL bad”. 

#### 3.3.5. Disruption to Daily Life.

In addition to reporting perceived health effects, users indicated how vaping disrupted their everyday life. Some users described feeling embarrassed because their clothes smelled after vaping and worried that other students or teachers would detect it. Others noted how vaping was impacting their physical functioning and performance in athletics. One user explained, “When I started sports here, we have to run a mile at cheer practice at the beginning and at the end. I feel like I started running slower and my breathing has gotten really worse, so I think I really should stop (vaping)”.

Notably, non-users reported that other student’s vaping behavior impacted their daily life, as well. In addition to feeling bothered by second-hand vape due to potential health effects and unpleasant smell, non-users did not like vapor blown in their face or peers vaping in class. Despite this, most reported that they do not ask others to stop and reported changing their own behavior to avoid confrontation. An underclassman explained, “If I walk in (the bathroom) and there’s a group vaping, I just walk out and walk an extra three miles just to go”.

#### 3.3.6. Use as a Coping Strategy 

Some students reported using JUUL as a stress relief. An upperclassman explained, “It’s a great stress reliever. I get anxiety attacks, so I go into the bathroom and I take a few hits, and then I’m fine… It’s kind of just something to ease my mind, but that’s the most that I use it for”.

A middle school participant reported using to cope with a difficult life situation. “The reason I started was some bad things going on. My parents divorced and it helped me calm down”.

### 3.4. Social Factors 

Themes in this category included peer and parental influences, lack of social support for quitting, and social accessibility.

#### 3.4.1. Peer Influences

Participants had a range of perspectives on peer influences. Some were adamant that teens pressured others to use JUUL. Others acknowledged that offering to share devices was common, but did not view this as pressure. Some participants suggested the prevalence of JUUL could put indirect pressure on teens to vape. These differing perspectives were present even among users. An underclassman explained how peer pressure influenced use, “…the reason I use is peer pressure…everybody saying, ‘Here. You should do that’”. 

The most common motivation for using JUUL was to “look cool,” suggesting a level of peer involvement is involved in the decision to try. Several non-users reported JUUL was less popular among their immediate friend group compared to their broader peer network. “None of my friends, like my close friends—they don’t really like that. ‘Cause I don’t want to be friends with someone that JUULs”. 

#### 3.4.2. Parental Influence 

Although most reported that their parent(s) would be a trusted source of information about vaping, only a few (users and non-users) reported conversations with their parents about it. Some had discussed cigarette smoking with their parents, but not e-cigarettes. Most anticipated they would be punished if their parents knew they used JUUL, with middle school students anticipating the most severe consequences. Some participants assumed that their parents would be disappointed, but would not punish them. A few users reported lenient parent rules, indicating their parents were indifferent to JUUL use. A Junior/Senior user said, “My parents are highly against me smoking, but they’re fine with me vaping”. 

#### 3.4.3. Lack of Support for Quitting

Participants noted a lack of support for quitting for adolescents. While some participants thought a trusted adult, such as a parent, coach, or teacher could help an adolescent quit, others thought that adolescents do not get support from adults or peer groups. A high school non-user explained, “They (users) don’t have nowhere to turn. If they go to their parents, they get punished. If they go to friends, they are vaping and they ask, ‘Why are you quitting?’”. 

Similarly, a few participants noted that tangible support including quit aids, like nicotine patches, are not available to adolescents. Participants recommended providing more concrete evidence about the health effects of using e-cigarettes to help adolescents quit. Some participants described their generation as “test subjects” and drew parallels between previous generations’ lack of understanding of the effects of smoking cigarettes. 

#### 3.4.4. Social Accessibility

Participants reported that JUUL was socially accessible and it was easy to obtain. Users routinely shared JUULs at school, parties, and athletic events. High schoolers reported that underage students asked 18-year-old peers, who were legal age to purchase tobacco in NC at the time of the study, to purchase JUUL for them and knew peers who sold products to middle school students, often for a more expensive price. 

One underclassman user explained, “It’s very readily accessible—I think it’s ‘cause kids in high school are 18. You see them every day, so it’s like, ‘Hey, can you get this for me and give it to me tomorrow when I see you in class.’”. 

Participants in focus groups at the schools in higher socioeconomic areas noted that students often own multiple JUULs, while students at schools in lower socioeconomic areas said students often share products with peers instead of owning one. 

### 3.5. Environmental Factors 

Themes in this category are related to school or community-level influences including school norms, contradictory messages about e-cigarettes, and lack of resources and services. 

#### 3.5.1. School Norms

Most high schoolers reported students using e-cigarettes “every day” or “always”. Use was reported “everywhere” at school, including classrooms, bathrooms, and hallways. They also observed that use seemed more common compared to their middle school years. A participant from the freshmen/sophomore group said, “I definitely heard about it in middle school, but I didn’t have the same experience with people doing it in the bathrooms or in class”. 

#### 3.5.2. Lack of School Policy Enforcement

Participants reported a range of consequences for using e-cigarettes at school. Middle school students described the most severe consequences. Participants from all schools reported that it is easy to vape at school and consequences vary depending on the teacher, ranging from no consequences to suspension or expulsion. Some reported that teachers know students are using at school but do not confiscate products or report students. For some, this lack of enforcement demonstrated a disregard for students’ health. A freshmen/sophomore user explained, “It depends on if you’re with a teacher that’s a pushover. They’re like, ‘Hey, you shouldn’t do that,’ but they won’t take it away”. 

Participants expressed their desire for teachers to enforce existing school policies on tobacco use. A freshmen/sophomore non-user questioned, “Do something about it because if you know the risks of being addicted to nicotine, why not take action into doing something?”. 

#### 3.5.3. Contrasting Messaging About e-Cigarettes

Participants reported receiving mixed messages about the health effects of using e-cigarettes from TV commercials, radio, and YouTube, as well as messages from social media influencers. The mixed messaging represents a stark contrast to anti-smoking messaging they reported is ingrained in their generation. A freshmen/sophomore non-user explained, “…we all grew up with the, ‘Don’t smoke cigarettes message’, I think that’s a pretty common thing for early 2000-borns”. 

#### 3.5.4. Lack of Health Education and Screening for e-Cigarette Use 

Adolescents did not consistently report receiving information about e-cigarette use from their doctors, teachers, coaches, or other adults. Participants indicated that they were not routinely screened for e-cigarette use by medical professionals. A Junior/Senior user said: “When I go to the doctor, they ask “Do you smoke?” But they don’t ask any sort of questions (about vaping)”.

## 4. Discussion

Adolescent health behavior is influenced by personal characteristics, peer and familial relationships, and distal factors such as school policies and neighborhood contexts [24]. Understanding these contexts informs prevention strategies that can be applied at multiple levels, which has shown to be most effective in changing behaviors [24]. Our study qualitatively explored middle and high school student perceptions of factors across social ecological levels that contribute to JUUL use and can be used to inform comprehensive approaches to preventing use. 

Consistent with previous research, participants in our study reported that e-cigarettes are appealing due to their novelty, discreteness and youth-friendly, non-traditional flavors, such as fruit, candy and sweets [25]. Participants reported previous use of the JUUL flavors that were voluntarily removed from retail stores including mango, crème, and cucumber [26,27], but indicated they had switched to mint-flavored pods, began using knock-off JUUL pods, or refilling the pods with a non-traditional flavored e-liquid. Adolescent use of the non-traditional flavors is associated with greater odds of continued vaping as well as a progression to vaping more frequently [14]. Thus, it is concerning that adolescents are willing to adapt new strategies to continue non-traditional flavored use and highlights that the US Food and Drug Administration’s (FDA) enforcement policy on unauthorized flavored cartridge-based e-cigarettes, which prioritizes removal of pod-based e-cigarettes in flavors other than tobacco and menthol, may not reduce adolescent e-cigarette use [27]. Further research is needed to determine if users will switch to menthol pods, a similar flavor to mint, switch to disposable vapes or tank systems, which are not included in the enforcement action, or refill existing pods with another flavored e-liquid brand.

Although participants identified potential long-term health effects, most groups discussed how there are unknown long-term health effects of using JUUL. Notably, users discussed more acute effects than non-users including coughing and headaches, which are similar to symptoms reported in the literature [28]. In addition, users also discussed eye irritation and nausea, which are associated with e-cigarette use [29,30]. Youth prevention campaigns may consider focusing on these acute health effects experienced by users. 

Most users described their first use negatively, recalling coughing, burning in the throat, and an element of surprise that nothing alerts the user to stop inhaling. While several studies have assessed subjective initial experiences of tobacco smoking and found it described as unpleasant with symptoms such as nausea and headaches [31,32], the few that have assessed first experiences with e-cigarettes found that few adolescents report negative symptoms [33]. Users in our study reported aversive initial use experiences, perhaps because they were describing JUUL specifically, which contains some of the highest nicotine levels on the market. Future studies should assess the trajectories of JUUL users compared to lower nicotine content e-cigarettes to determine if there are differences in progression and sustained use of e-cigarettes and/or combustible tobacco products. 

Finally, adolescents reported impacts of vaping on their everyday life. Some users indicated they worried that teachers or peers would discover they vaped, while others described its impact on physical functioning, noting how vaping had adversely affected their respiratory health, and thus their reduced performance in activities. Further, some users reported symptoms of dependence and described loss of control. These findings indicate that some users not only recognize the impact of vaping on their mental and physical health, but are able to connect it to an impact on their daily life, such as reduced participation in sports or feelings of embarrassment, worry and loss of control. More research is needed to better understand adolescent perceptions of how vaping impacts their daily life. Interventions, such as media campaigns, could focus on these more relatable consequences, much like the FDA’s The Real Cost campaign did in focusing on loss of independence and addiction for cigarette smoking [34]. 

Notably, non-users also reported that vape impacts their daily life, primarily at school, and described changing their behavior to avoid those vaping. These findings highlight the need to enforce school vaping policies, not only to reduce use, but to safeguard students who are not using.

### 4.1. Social Influences

Peer use is a common reason for use cited for youth and young adults [11,34,35,36]. We found students were mixed on the influence of peers, with some attributing their use to peer pressure while others downplaying its affect. Some non-users indicated they selected peers who do not use, suggesting adolescents may insulate themselves from e-cigarette use by being selective about friend groups and choosing not to be with adolescents who use. Peers can also help students build skills to resist peer pressure and a resource for students who need social support to quit vaping [37]. 

Parents also play an important role adolescent tobacco use [38,39]. Although some participants reported parental discussions about smoking cigarettes, most indicated that they had not discussed vaping. Students indicated parents would be a trusted source of information, suggesting a missed opportunity for health education. However, most parents are unaware of JUUL or its high nicotine content [39]. Thus, parents need reliable sources so they can be best situated to provide up-to-date information to their children about the risks. To fill this gap, schools could take the lead in alerting parents to new trends, with help from pediatricians and public health officials. 

### 4.2. Environmental Factors

Schools provide an ideal setting to reach adolescents and implement a comprehensive approach to tobacco prevention. [40] A key component is a clearly communicated tobacco-free policy that is applied and enforced to reduce use among adolescents [41]. Both users and non-users reported a lack of school policy enforcement, which could be due to several reasons. First, school staff could lack knowledge about the products. In a recent study, less than half of school staff correctly identified JUUL in a photo, suggesting staff may need training product identification [42]. Additionally, evidenced-based and theory-informed curriculum for tobacco prevention and education, such as the Tobacco Prevention Toolkit, could be incorporated, further assisting schools in addressing the problem [43]. However, the lack of reporting could also be due to the policy and its stated consequences. The punishment may be perceived as too severe by school staff, resulting in a lack of reporting students who are vaping at school. Thus, a review of the school policy with administrators, school staff, parents and students may be warranted to ensure the students receive the resources needed and the consequences are not perceived as too harsh. 

Participants also noted contradictory messages about the health effects and safety of e-cigarettes, which is not surprising since public health campaigns aimed at adolescents have focused on the negative effects of e-cigarettes, while messages aimed at adult smokers have highlighted the benefits of e-cigarettes [44,45]. More research is needed to understand how conflicting messages about e-cigarettes are interpreted by adolescents and the effect it has on their behavior. 

Adolescents reported a lack of health education provided by adults, most notably health care providers. The American Lung Association and the American Academy of Pediatrics recommend that health care organizations add questions specific to e-cigarette use to the Electronic Health Record so that use and associated consequence can be tracked over time [46,47]. However, few healthcare systems have discrete fields for e-cigarette documentation and clinicians report little training on documentation of e-cigarette use [48]. Given that e-cigarettes are the tobacco product of choice among teens, these challenges must be addressed to incorporate vape-specific questions into health screenings.

## 5. Conclusions

Findings highlight factors at multiple levels contributing to JUUL use among middle and high school students. Targeted interventions are needed to help students resist use and help them quit. Potential strategies include increasing adolescent knowledge of potential harms through evidence-based curricula and communication campaigns; providing counseling and cessation resources; reviewing existing policies; and training school officials to identify products.

### Limitations

Only one of the six groups was conducted at a middle school, making middle versus high school comparisons limited. For some topics (e.g., addiction, use patterns), non-users reported secondhand information based on their observations. Given the sensitive nature of the topic, their perspective still provides meaningful insight. Finally, because the e-cigarette environment changes rapidly, our findings do not reflect the influence of EVALI or new disposable products, such as Puff Bar, on adolescent use and perceptions.

## Figures and Tables

**Table 1:** Sample characteristics.

	User	Non-User	Total
	*n* = 13	*n* = 16	*n* = 29
	Mean (SD)	Mean (SD)	Mean (SD)
Age	16.8 (1.3)	15.1 (1.5)	15.9 (1.6)
Gender	Count (%)	Count (%)	Count (%)
Male	5 (38.5)	7 (43.7)	12 (41.4)
Female	8 (61.5)	9 (56.3)	17 (58.6)
Race			
African American	1 (7.7)	4 (25)	5 (17.2)
Caucasian	10 (76.9)	2 (12.5)	12 (41.4)
Asian	-	1 (6.3)	1 (3.4)
2+ reported	2 (15.4)	1 (6.3)	3 (10.3)
Ethnicity			
Hispanic or Latino	-	8 (50)	8 (27.6)
Grade Level			
Middle School (6–8)	-	5 * (31.3)	5 (17.2)
High School (Fr/So)	3 (23.1)	9 * (56.3)	12 (41.4)
High School (Jr/Sr)	10 (76.9)	2 (12.5)	12 (41.4)

* Student reported no use on the screener, but identified as an ever user during the focus group and on the Participant Survey.

**Table 2:** Major themes.

Individual Factors	Similarities/Differences by User Status
Flavors and impact on use	Flavors contribute to adolescent use (U, N). Users reported strategies to use flavors when pods were no longer available.
* First experience with JUUL	Users reported negative first experience with JUUL, but continued use (U).
Perceived addiction	JUUL is addictive; Know someone addicted (U, N). Some users reported symptoms of addiction.
Risk Perceptions	Potential health effects including cancer, lung problems and unknown effects (U, N). Users reported more acute health effects (i.e., wheezing, headache).
Disruption to daily life	Feelings of embarrassment and worry that others will find out about their vaping; Recognition that vaping impacts physical functioning (U).Bothered by second-hand vape; Change daily routine to avoid vapers (N)
* Use as coping strategy	Some reported use to ease anxiety (U).
**Social Factors**	
Peer and Parental Influence	Peers can influence use and non-use (U, N); Parents are a trusted source of information (U, N).Limited parent/child conversations about vaping (U, N)
Lack of support for quitting	Lack of social support and cessation aids (U, N).
Social accessibility	Easy to obtain and users share devices (U, N).
**Environmental Factors**	
School norms	Use is common at school (U, N). Non-users reported being bothered by secondhand vapor and some changed behavior to avoid those vaping (N).
Lack of school policy enforcement	Easy to vape at school (U, N); Consequences are school- and teacher-dependent (U, N). Non-users want more school policy enforcement (N).
Conflicting e-cigarette messages	Mixed messages about health effects for e-cigarettes (U, N).
Lack of health education and screening	No consistent health education or screening for vaping (U, N).

* Questions only asked of users; U = User; N = Non-User
